# Fever, pancytopenia, and elevated D-dimer in a 95‐year‐old woman with ehrlichiosis: a case report

**DOI:** 10.1186/s12877-021-02129-6

**Published:** 2021-03-12

**Authors:** Christopher Radcliffe, Cynthia Tsay, Kimberly Glerum, Jane Liao, George Goshua, Gerard Kerins

**Affiliations:** 1 Yale University School of Medicine, 333 Cedar St., CT 06510 New Haven, USA; 2 Department of Internal Medicine, Yale University School of Medicine, New Haven, CT USA; 3 Section of Hematology and Oncology, Department of Internal Medicine, Yale University School of Medicine, New Haven, CT USA; 4 Section of Geriatric Medicine, Department of Internal Medicine, Yale University School of Medicine, New Haven, CT USA

**Keywords:** Ehrlichiosis, Pancytopenia, Pulmonary embolus, Case report

## Abstract

**Background:**

Pancytopenia, fever, and elevated D-dimer are significant clinical findings. The differential diagnosis includes hematological malignancies, severe coronavirus disease 2019 (COVID-19), tick-borne illnesses, and other etiologies.

**Case presentation:**

We report the case of a 95-year-old woman who presented with high fever (103.6 °F), pancytopenia, and markedly elevated D-dimer (32.21 mg/L; reference range ≤ 0.95 mg/L) in late-autumn during the COVID-19 pandemic at a large academic institution. After remaining persistently febrile, a peripheral blood smear was ordered and revealed parasites consistent with *Ehrlichia *spp. Doxycycline monotherapy led to symptomatic improvement and resolution of her pancytopenia. During her hospital stay, a computed tomography angiogram of the chest revealed pulmonary emboli, and esophagogastroduodenoscopy uncovered arteriovenous malformations. After appropriate treatment, she was discharged on hospital day 7 and has since done well.

**Conclusions:**

Overall, our case offers a dramatic, unexpected presentation of ehrlichiosis in a nonagenarian. To our knowledge, this is the first report of concurrent ehrlichiosis and pulmonary embolus.

## Background

Fever, pancytopenia, and elevated D-dimer are significant clinical findings in an elderly adult. The differential diagnosis is broad; however, the current pandemic has led to a widespread focus on coronavirus disease 2019 (COVID-19) and its range of clinical presentations. We report the case of a 95-year-old woman who presented with altered mental status, fever, pancytopenia, and markedly elevated D-dimer. After repeatedly testing negative for severe acute respiratory syndrome coronavirus 2 (SARS-CoV-2), a targeted approach led to an unexpected diagnosis: pulmonary emboli with concurrent ehrlichiosis.

Human monocytotropic ehrlichiosis is a tick-borne infection caused by members of the genus *Ehrlichia*, and it classically presents as a febrile illness with headache, systemic symptoms, and hematologic abnormalities [1]. Pancytopenia is rarely reported [2, 3]. To our knowledge, we report the first case of pulmonary emboli in association with ehrlichiosis. Notably, the patient remained asymptomatic throughout her hospital course and was safely discharged home. Our case reinforces the principle that older adults often have atypical presentations of typical diseases.

## Case presentation

A 95-year-old woman with a past medical history of iron deficiency anemia, asthma, colorectal adenocarcinoma status-post hemicolectomy, and collagenous colitis presented in late autumn with altered mental status during the COVID-19 pandemic. She had been social distancing at her daughter’s home in the northeastern United States and was brought to medical attention due to weakness, confusion, and an unresponsive episode in the days preceding admission.

On presentation, she denied all symptoms and was only oriented to self. At baseline, she had no evidence of cognitive impairment and was able to complete activities of daily living. On admission, vital signs were notable for intermittent tachycardia, tachypnea, and high fever (103.6 °F, rectal). On exam, she was anxious appearing and found to have diffuse expiratory wheezing. No rashes were noted.

In the emergency department, serum studies revealed low hemoglobin (9.2 g/dL, baseline 11.8 g/dL; reference range 12.0–18.0 g/dL) and new thrombocytopenia (112 × 10^3^/µL; reference range 140–440 × 10^3^/µL). A computed tomography scan of the head and a chest radiograph were unrevealing. SARS-CoV-2 RNA and other respiratory viruses were not detected on a nasopharyngeal specimen. Peripheral blood cultures were obtained, and she was empirically started on vancomycin and piperacillin-tazobactam.

**Fig. 1:**
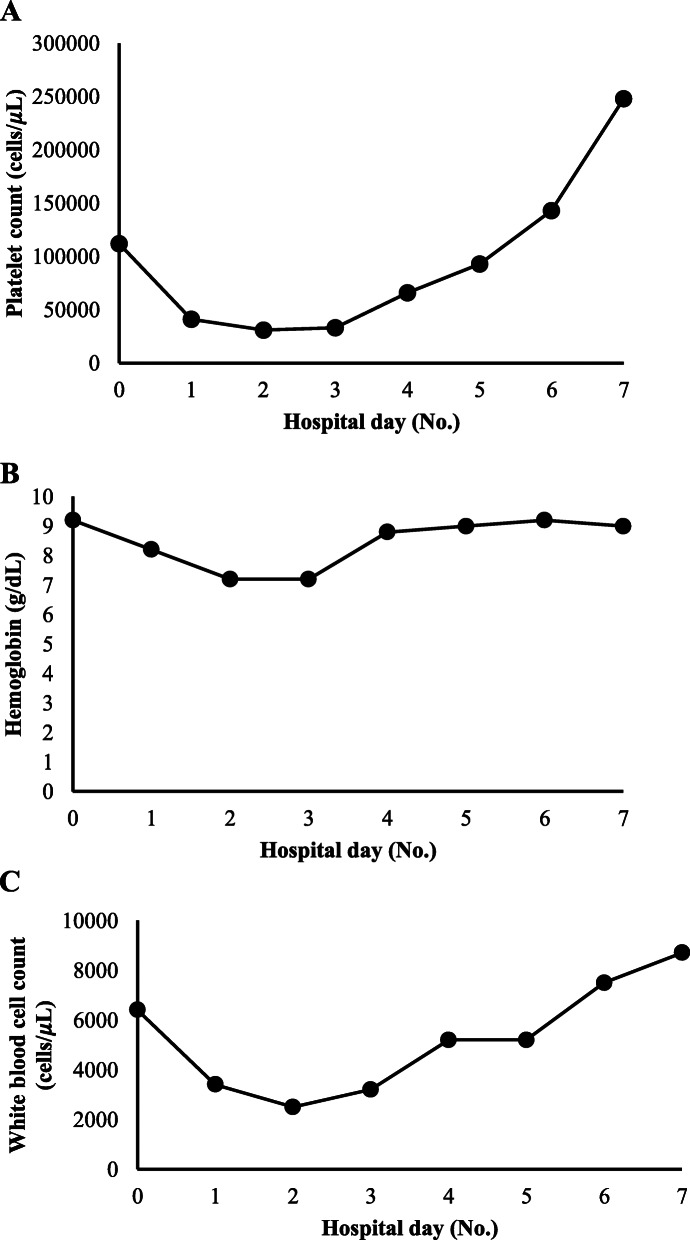
Cell counts during hospital course. Plots for cell counts are depicted in panels (**a**) platelet count (reference range 140–440 × 10^3^/µL), (**b**) hemoglobin (reference range 12.0–18.0 g/dL), and (**c**) white blood cell count (reference range 4.0–10.0 × 10^3^/µL). Hospital day zero on the x-axis denotes laboratory measurements obtained on admission

On hospital day one, dropping hemoglobin (8.3 g/dL), new leukopenia (3.2 × 10^3^/µL; reference range 4.0–10.0 × 10^3^/µL) with reduced absolute lymphocyte count (300/µL; reference range 1.0–4.0 × 10^3^/µL), and worsening thrombocytopenia were found (43 × 10^3^/µL). Figure 1 summarizes cell count measurements obtained during the hospital course. Additionally, a markedly elevated D-dimer level was uncovered (32.21 mg/L; reference range ≤0.95 mg/L). Given continued suspicion for SARS-CoV-2 infection, a repeat nasopharyngeal specimen was obtained and returned negative. Antimicrobial therapy was narrowed to cover for community acquired pneumonia.

At this point, the diagnostic approach was framed around finding a unifying reason for new pancytopenia, fever, and elevated D-dimer. The index of suspicion was initially high for SARS-CoV-2 infection given the lymphopenia, fever, and elevated D-dimer along with ongoing wheezing and tachypnea; [4, 5] however, two negative tests made this less likely. In general, pancytopenia can result from infectious etiologies, infiltrative metastases, nutritional deficiencies, autoimmune conditions, hematological malignancies, or drug reactions [6]. The differential diagnosis for elevated D-dimer overlaps with that of pancytopenia and includes malignancy, venous thromboembolism, advanced age, and infection [7].

Our patient’s high fever and acute presentation prompted infectious causes to be considered. In the absence of immunosuppressive medications or active malignancy, her immune status was only compromised by her advanced age. She lacked a significant travel history, and the differential diagnosis for infectious causes of pancytopenia was narrowed to bacterial sepsis versus tick-borne illness. Upon obtaining further history from the patient and her daughter, it was noted that she had been exposed to several dogs at home, and they were known to have ticks.

Given her new thrombocytopenia and leukopenia accompanied by persistent fevers, a peripheral blood smear was ordered, and parasites consistent with *Ehrlichia* spp. were identified. The elevated D-dimer was multifactorial in the setting of advanced age and systemic infection, but a pulmonary embolus was possible and she intermittently required supplemental oxygen. A computed tomography angiogram of the chest was ordered and revealed right segmental pulmonary emboli. D-dimer (5.37 mg/L) level remained elevated on hospital day two.

After finding the acute pulmonary emboli, an inferior vena cava (IVC) filter was placed by interventional radiology, and a heparin infusion was started the following day. During her treatment course, an episode of melena and need for ongoing anticoagulation prompted an esophagogastroduodenoscopy which revealed small arteriovenous malformations that were treated with ablation. No episodes of melena occurred during the remainder of her hospital stay.

By hospital day 7, her platelet (248 × 10^3^/µL) and white blood cell (8.7 × 10^3^/µL) counts had normalized, and her anemia had improved (hemoglobin 9.0 g/dL). She was discharged home on doxycycline and apixaban. Two weeks later, she was seen in an out-patient setting and had been doing well.

## Discussion and conclusions

We report the case of a 95-year-old woman who presented with altered mental status, high fever, and pancytopenia. Surprisingly, she denied any symptoms and repeatedly voiced her desire to return home. Clinical suspicion for COVID-19 was high; however, a peripheral blood smear clinched the diagnosis, and further history provided a plausible source of infection – ticks from dogs. Doxycycline monotherapy led to normalization of cell counts by the time of discharge.

In general, pancytopenia is a rare manifestation of ehrlichiosis, [2, 3] and pancytopenia has also been reported with other tick-borne illnesses like Lyme disease [8] and anaplasmosis [6, 9]. Notably, co-infection with multiple tick-borne pathogens (e.g. *Babesia* spp. and *Borrelia burgdorferi*) can lead to severe disease [10]. Despite our patient’s advanced age, improvement in cell counts was achieved after selection of appropriate antimicrobial therapy.

To our knowledge, this is the first report of a pulmonary embolus complicating the clinical course of a patient with ehrlichiosis, and the temporal association was striking. Interestingly, pulmonary emboli and other vascular phenomena have been reported in association with neoehrlichiosis caused by *Candidatus* Neoehrlichia mikurensis, [11] and varied forms of vasculitis were identified in a small case series on patients with serological evidence of ehrlichiosis [12]. Our patient had multiple risk factors for development of pulmonary emboli, so it is unclear whether direct vascular inflammation from the infection contributed to her presentation and supportive histopathological evidence is not available for our case. Nonetheless, numerous viral and bacterial pathogens have been implicated with vasculitis, and the tropism of *Ehrlichia chaffeensis* is known to include endothelial cells [13]. Further exploration of the association between ehrlichiosis and vascular inflammation may be warranted.

This case provides an atypical, dramatic presentation of ehrlichiosis in a 95-year-old woman. Severe laboratory derangements were concerning, and her treatment course was notable for both pulmonary emboli and a gastrointestinal bleed. Nonetheless, she denied symptoms, and this reinforces the principle that older adults experience systemic illness differently than their younger counterparts. Our patient was independent in her activities of daily living and was able to be safely discharged home. The risk-benefit discussion behind choosing to place an IVC filter was guided by her baseline functional status, and device retrieval is anticipated to happen in the coming months. Overall, our case highlights a rare presentation of ehrlichiosis in a 95-year-old woman and emphasizes the need to remain vigilant for atypical presentations in geriatrics.

## Data Availability

All relevant, deidentified data has been presented in the manuscript and further inquiry can be directed to the corresponding author.

## Abbreviations

COVID-19: Coronavirus disease 2019
IVC: Inferior vena cava
SARS-CoV-2: Severe acute respiratory syndrome coronavirus 2
